# *KMT2A* Mutations and High Prevalence of dMMR-associated Mutational Signatures as Prognostic Indicators in Metastatic Colorectal Cancer

**DOI:** 10.7150/jca.94410

**Published:** 2024-04-08

**Authors:** Zhihang Han, Chuanjun Song, Dongqing Li, Weiyou Zhu, Jiukang Sun, Jialing Yao, Wenyuan Gan, Fufeng Wang, Xiaodong Yang, Lingjun Zhu

**Affiliations:** 1 The First Affiliated Hospital of Nanjing Medical University, No. 300 Guangzhou Road, Nanjing, Jiangsu Province,210029, China.; 2 Xinghua People's Hospital Affiliated to Yangzhou University, No. 419 Yingwu South Road, Xinghua, Jiangsu, 225700, China.; 3 Collaborative Innovation Center for Cancer Personalized Medicine, Nanjing Medical University, Nanjing, Jiangsu Province, 210029, China.; 4 Nanjing Geneseeq Technology Inc, Nanjing, Jiangsu Province, 210018, China.

**Keywords:** colorectal cancer, prognostic indicators, next-generation sequencing, KMT2A mutations, dMMR-associated Mutational Signatures

## Abstract

The conventional treatment strategies for patients with metastatic colorectal cancer (mCRC) are predominantly guided by the status of RAS and BRAF mutations. Although patients may exhibit analogous pathological characteristics and undergo similar treatment regimens, notable disparities in their prognostic outcomes can be observed. Therefore, tissue and plasma samples from 40 mCRC patients underwent next-generation sequencing targeting 425 cancer-relevant genes. Genomic variations and canonical oncogenic pathways were investigated for their prognostic effects in association with progression-free survival (PFS) of these patients. We found that patients with *BRCA2* and *KMT2A* mutations exhibited worse prognostic outcomes after chemotherapy-based treatment (univariate, P < 0.01). Further pathway analysis indicated that alterations in the homologous recombination pathway and in the *KMT2A* signaling network were also significantly associated with shortened PFS (univariate, P < 0.01). Additionally, mutation signature analysis showed that patients with higher proportions of defective mismatch repair (dMMR)-related mutational signatures. Had a worse prognosis (univariate, P = 0.02). *KMT2A* mutations (hazard ratio [HR], 4.47; 95% confidence interval [CI], 1-19.93; P =0.050) and dMMR signature proportions (HR, 3.57; 95% CI, 1.42-8.96; P = 0.007) remained independently associated with PFS after multivariate analysis and the results were further externally validated. These findings may enhance our understanding of this disease and may potentially facilitate the optimization of its treatment approaches.

## Introduction

Colorectal cancer (CRC) ranks as the third most prevalent and the second most lethal malignancy worldwide. The primary cause of mortality in CRC patients is metastasis, which significantly diminishes the prognosis of those affected 1. Despite advancements in treatment modalities, the five-year survival rate for patients diagnosed with metastatic CRC (mCRC) remains dismally low, ranging between 11% and 15% 2. There is an urgent need for the identification and validation of robust prognostic biomarkers that could pave the way for early intervention and broaden the spectrum of therapeutic options for mCRC patients.

The prognostic landscape of CRC has traditionally relied on clinicopathological characteristics, with the stage at diagnosis serving as a pivotal indicator of patient outcomes. Other factors influencing prognosis include tumor localization, the presence of perineural invasion, and the degree of histological differentiation 3. In recent years, the advent of molecular biology, alongside breakthroughs in immunotherapy and targeted treatments, has revolutionized the treatment landscape for advanced cancers. This paradigm shift has sparked a growing interest in the exploration of molecular biomarkers as tools for prognostication in CRC.

Microsatellite instability (MSI) is a favorable prognostic factor in stage II CRC but may have a marginal negative impact on survival in the metastatic setting 4-6. The presence of mutations in the RAS and BRAF genes plays a critical role in determining treatment efficacy and thereby influencing prognostic outcomes in mCRC. Evidence from a meta-analysis encompassing five clinical trials illustrates the correlation between mutations in *KRAS* and *BRAF* and reduced progression-free survival (PFS) and overall survival (OS) rates 7. In addition, the CpG island methylator phenotype (CIMP), characterized by aberrant methylation patterns, is associated with a shorter OS and varying responses to conventional chemotherapy 8, 9. The co-occurrence of KRAS/BRAF mutations or MSI in CIMP-positive tumors complicates the assessment of CIMP's independent prognostic value 10.

Despite these insights, the clinical adoption of prognostic biomarkers in CRC remains limited, underscoring the necessity for further investigation. Leveraging the advantage of next-generation sequencing, this study aims to delve deeper into the prognostic markers for CRC, with the objective of uncovering novel, independent prognostic indicators that could inform therapeutic decisions and enhance clinical outcomes for CRC patients.

## Material and Methods

### Patient and sample inclusion

This study retrospectively collected data from patients diagnosed with late-stage recurrent or metastatic colorectal cancer (mCRC) who received treatment at Jiangsu Provincial People's Hospital between November 2017 and February 2022. The patient selection criteria were as follows.

Inclusion criteria: 1) Diagnosis of late-stage recurrent or metastatic colorectal cancer. 2) Availability of comprehensive clinical information. 3) Detailed records of treatment received, including medication types, treatment duration, and progression-free survival data. (4) Availability of tumor tissue samples suitable for next-generation sequencing (NGS) analysis.

Exclusion criteria: 1) Incomplete clinical or treatment information. 2) Patients who underwent surgery after treatment.

According to these criteria, a total of 40 CRC patients were included in the study.

### Library preparation and sequencing

Hybridization-based target enrichment of 437 cancer-related genes was carried out using the GeneseeqPrime® pan-cancer gene panel with xGen Lockdown Hybridization and Wash Reagents Kit (Integrated DNA Technologies). Captured libraries by Dynabeads M-270 (Life Technologies) were amplified in KAPA HiFi HotStart ReadyMix (KAPA Biosystems) and quantified by qPCR using KAPA Library Quantification Kit (KAPA Biosystems). The final libraries were sequenced on Hiseq4000 platform (Illumina).

### Sequencing data and bioinformatics analysis

Trimmomatic was used for FASTQ file quality control. Leading or trailing low-quality bases (Q < 20) and N bases were removed 11. The sequencing data was aligned to the reference Human Genome (hg19) using Burrows-Wheeler Aligner (BWA-mem, v0.7.12) 12, and was then de-duplicated by Sambamba 13. Base quality recalibration and indel realignment were processed by Genome Analysis Toolkit (GATK 3.4.0) 14. VarScan2 15 was employed for calling single-nucleotide variations (SNVs) and insertion/deletions (indels). Genomic fusions were identified by FACTERA 16 with default parameters. Copy-number variations (CNVs) were detected using CNVkit 17 with default parameters. Somatic CNVs were identified comparing paired normal and tumor samples with the cut-off ratio of 0.6 for copy-number loss and 2.0 for copy-number gain. Pathway analyses referred to the STRING database (https://www.string-db.org/) for signaling networks.

### Mutation signature analysis

“Sigminer” package (https://cran.r-project.org/web/packages/sigminer/) was used to extract mutational signatures 18. The mutational patterns were compared with the COSMIC mutational signatures v3.1 reported by Alexandrov et al. 19. The contributions of the signatures sharing the same etiological origins were summed. For example, single base substitution (SBS) signatures 6, 14, 15, 20, 21, 26, and 44, all of which are associated with dMMR, were combined to represent the signature of dMMR.

### Statistical analysis

Log-rank tests were used to analyze PFS differences among groups. Multivariate analyses using the Cox proportional hazards model were performed to investigate the association between patients' survival and their clinical or genomic characteristics. A two-sided *P*-value of less than 0.05 was considered significant. All statistical analyses were performed in R (version 3.6.3).

## Results

### Patient Characteristics and Clinical Factors on Prognosis

**Figure 1:**
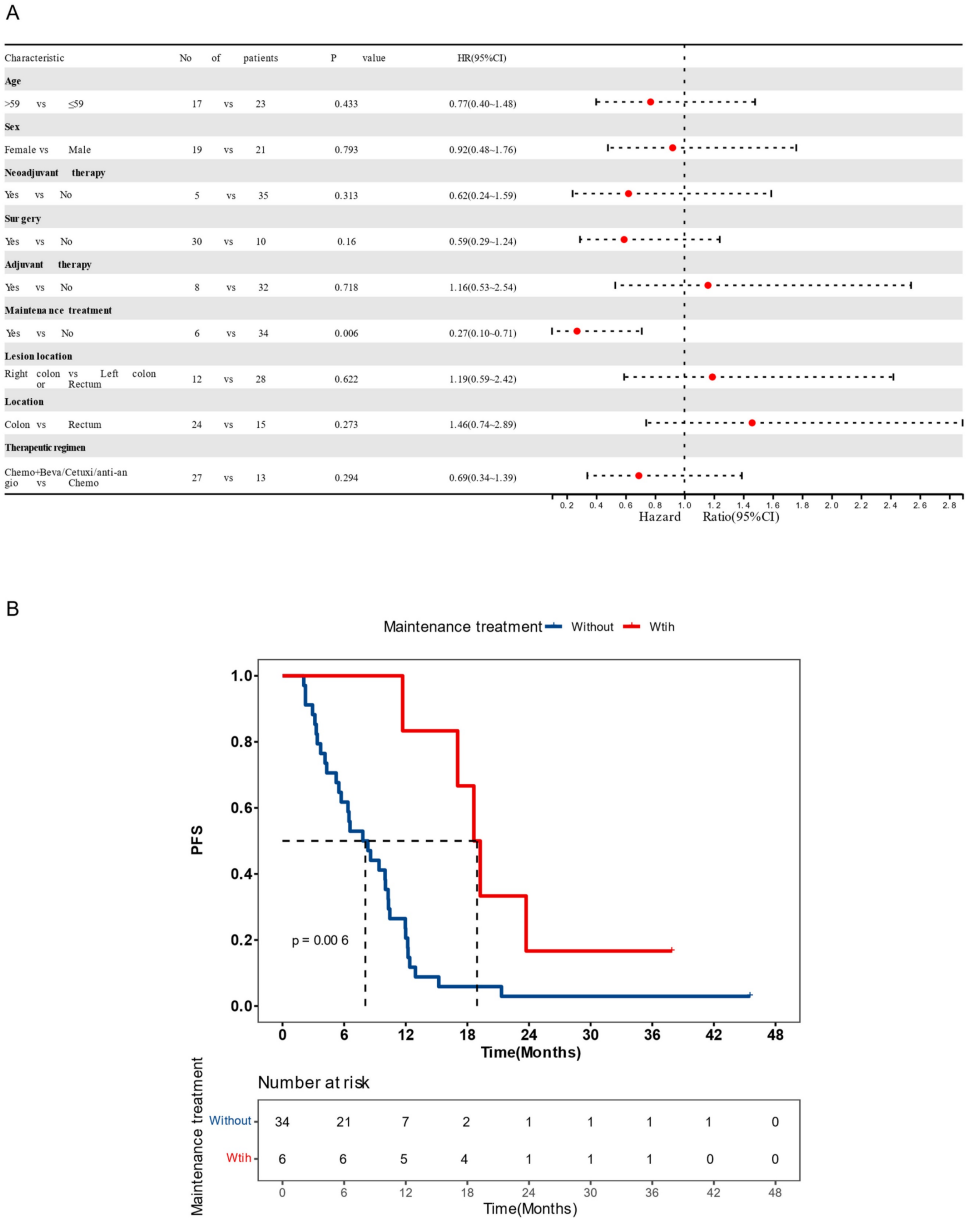
**Clinical features on prognosis** (A) Cox univariate analysis showing the impact of clinical features on patient progression-free survival (PFS). (B) Kaplan-Meier estimates comparing PFS of patients with or without maintenance therapy. Patients receiving maintenance therapy had significantly longer PFS (*P* < 0.05). Median PFS was indicated by dashed lines. anti-angio, anti-angiogenic therapy; Beva, bevacizumab; Cetuxi, cetuximab; CI, confidence interval; Chemo, chemotherapy; HR, hazard ratio.

A total of 40 CRC patients were included in the study (Figure s1, Table 1), with a median age of 59 (ranging from 44 to 82 years old). Among these patients, 21 were males (52.5%) and 19 were females (47.5%). The primary tumor sites were distributed as follows: left colon (n=12, 30%), right colon (n=12, 30%), rectum (n=15, 37.5%), and left colon plus rectum (n=1, 2.5%).30 (75%) patients had surgery history. All patients were classified as stage IV according to the American Joint Committee on Cancer (AJCC) criteria and received chemotherapy-based regions. The majority of patients (21, 52.5%) had received chemotherapy combined with bevacizumab, followed by 13 (32.5%) receiving chemotherapy alone, 5 (12.5%) receiving chemotherapy combined with cetuximab, and 1 (2.5%) receiving chemotherapy combined with anlotinib. The median PFS (mPFS) was 9.4 months and the median follow-up time was 13.7 months. Of all clinical factors, only administration of maintenance therapy was significantly associated with survival (Figure 1A and B) and was thus included in subsequent multivariate analyses.

### Identification of Prognostic Biomarkers

**Figure 2:**
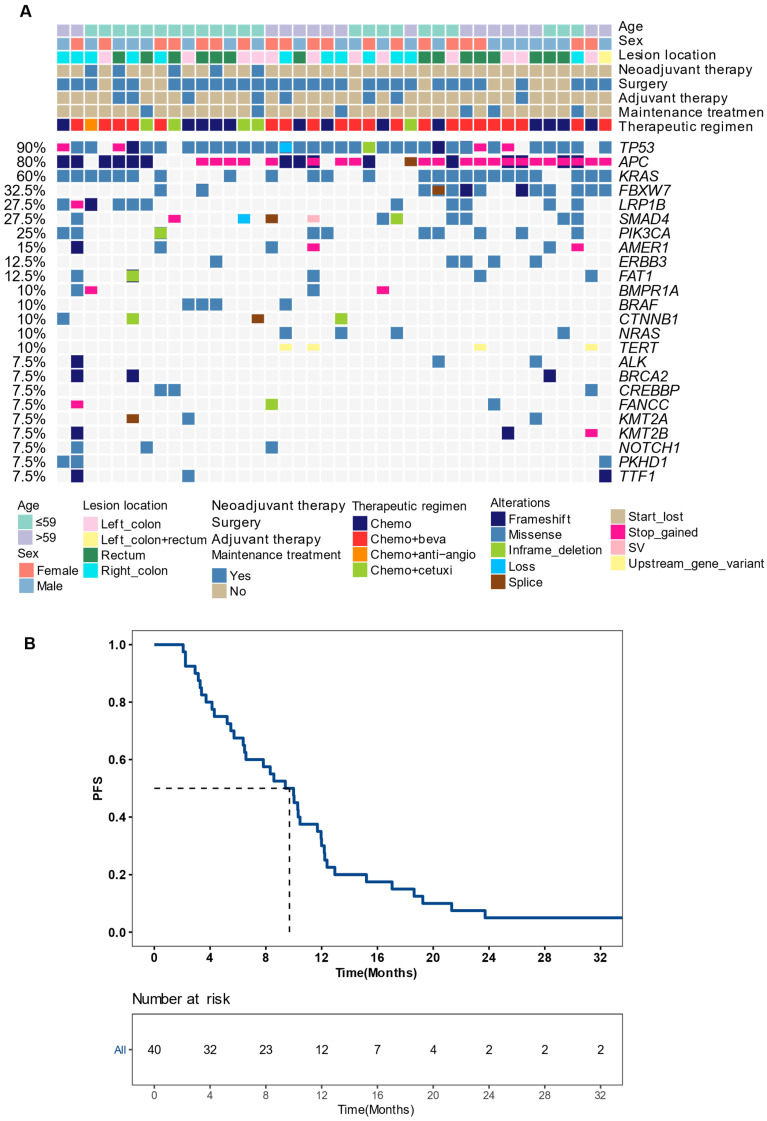
**Recurrence risk and mutation profiling of patients** (A) The mutational landscape of enrolled patients as detected from their baseline tissue samples. Genes with ≥5% mutation prevalence were displayed. (B) A Kaplan-Meier curve showing progression-free survival (PFS) of the studied population. Median PFS was indicated by dashed lines. anti-angio, anti-angiogenic therapy; beva, bevacizumab; cetuxi, cetuximab; Chemo, chemotherapy; SV, structural variant as gene fusion or rearrangement.

**Figure 3:**
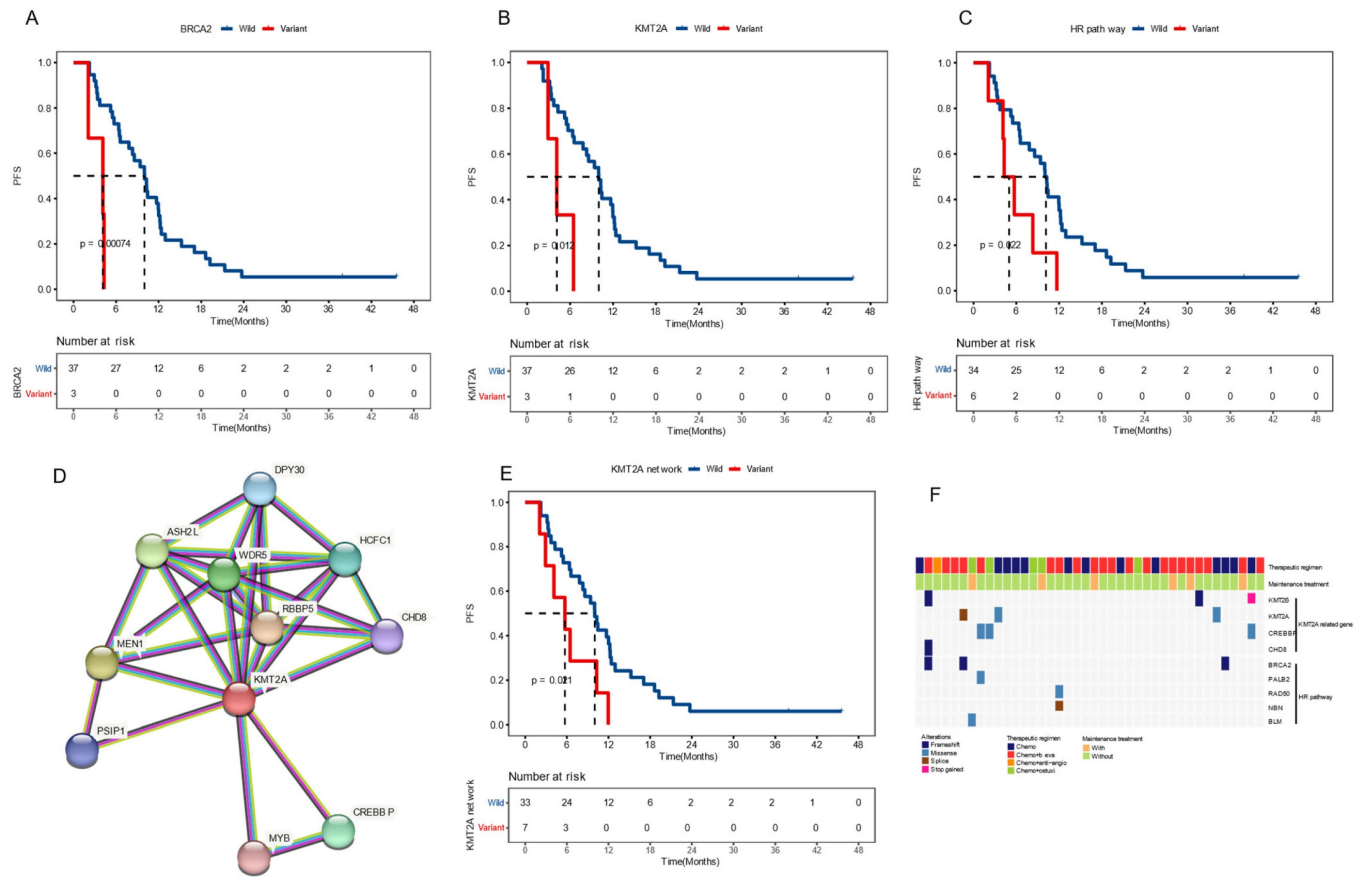
**Baseline molecular features on prognosis** (A-C) Kaplan-Meier plots of progression-free survival in patients with and without* BRCA2* mutations (A), *KMT2A* mutations (B), and altered homologous recombination (HR) pathway (C). (D) The signaling network associated with *KMT2A* as shown in the STRING database. (E) Kaplan-Meier plots of progression-free survival in patients with and without mutations in the *KMT2A*-related network. (F) The mutational profile of genes in the *KMT2A* network and the HR pathway. Median PFS was indicated by dashed lines. anti-angio, anti-angiogenic therapy; beva, bevacizumab; cetuxi, cetuximab; Chemo, chemotherapy.

To identify potential molecular biomarkers associated with outcomes, we profiled the mutational landscape of the studied population (Figure 2A). The top four mutated genes were *TP53* (90%), *APC* (80%), *KRAS* (60%), and *FBXW7* (32.5%), consistent with previous reports on CRC 20. Only genes that mutated in more than three patients were included in prognosis analysis. Our findings revealed that patients with *BRCA2* or *KMT2A* mutations had a worse prognosis. The mPFS for *BRCA2*-mutated (*BRCA2*+) patients was 4.1 months, compared to 10.0 months for those with wildtype *BRCA2* (P < 0.001), showing a hazard ratio (HR) of 7.30 with a 95% confidence level (CI) of 1.88-28.38. Similarly, patients with *KMT2A* mutations had a mPFS of 4.1 months, compared to 10.0 months for those without (*P* = 0.012, HR [95% CI]: 4.41 [1.23-15.82]) (Figure 3A-B). gene mutations were further grouped based on the signaling pathways or networks in which they are involved, including the top ten cancer-related pathways and the DNA damage response (DDR) pathways 21, 22 (Figure 3F). We found that mutations in the homologous recombination (HR) pathway genes were significantly associated with a poor prognosis, as patients with such mutations had a mPFS of 5.0 months compared to 10.2 months in those without (*P* = 0.022, HR [95% CI]: 2.81 [1.11-7.11]) (Figure 3C, 3F). Significant difference in PFS (5.7 vs 10.0 months) was also observed between patients with and without mutations in genes in the *KMT2A*-related signaling network (*P* = 0.021, HR [95% CI]: 2.68 [1.12-6.39]) (Figure 3D-F).

**Figure 4:**
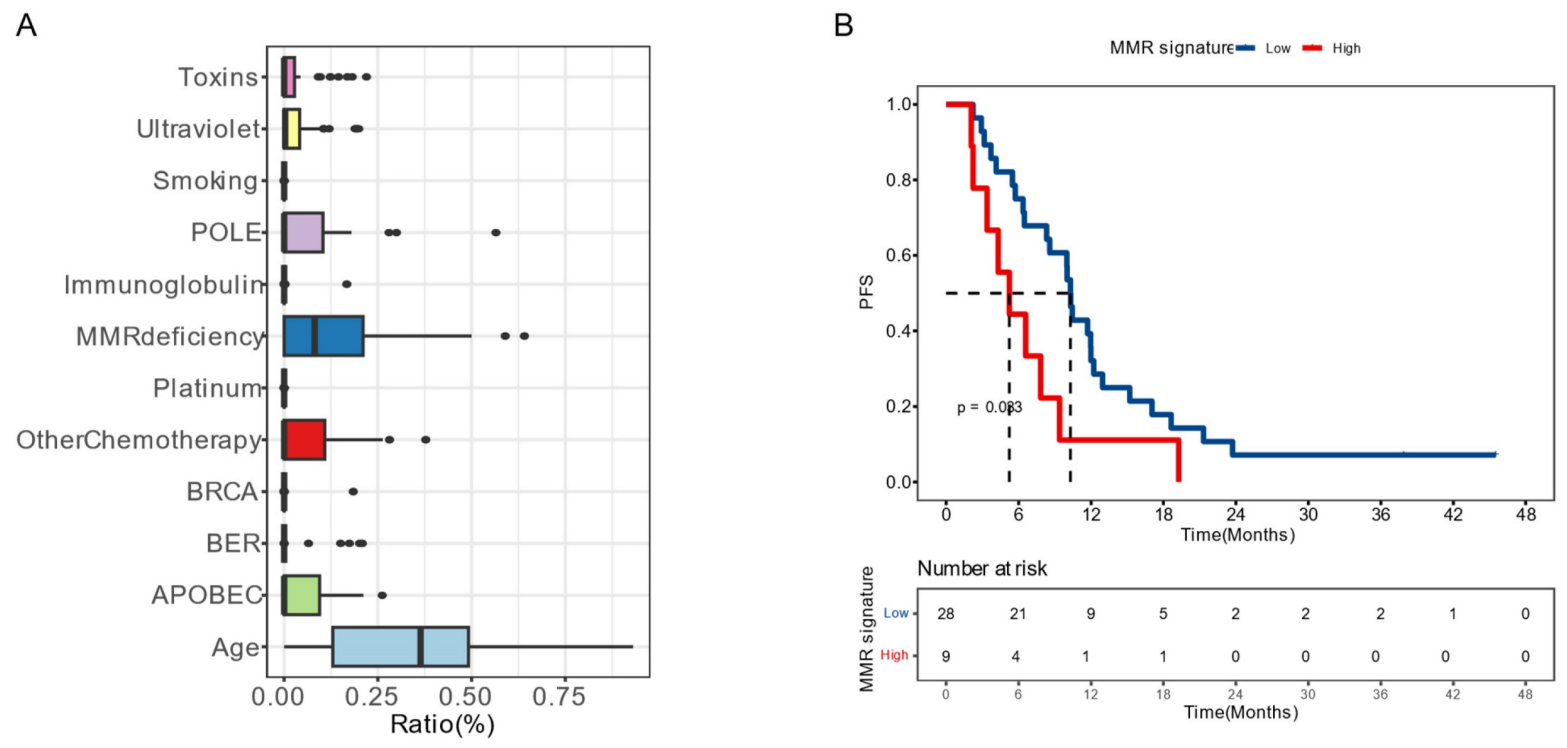
**Mutation signatures on patient prognosis** (A) Distribution of the mutation signatures clustered from COSMIC (v3.3) single base substitutions. (B) Kaplan-Meier plots of progression-free survival in patients with high or low proportion of deficient mismatch repair (dMMR) signature. Median PFS was indicated by dashed lines. BER, base excision repair.

Moreover, we examined the impact of mutational signatures on the prognosis of 37 patients. The mutational signatures in the cohort were predominantly influenced by age, dMMR, and activation-induced cytidine deaminase (APOBEC) (Figure 4A). We determined an optimized cut-off value for the proportion of dMMR-associated signatures at 1.17×10^-5^. Patients with a higher proportion of dMMR signature (>1.17×10^-5^) had a significantly worse prognosis, with a mPFS of 6.6 months compared to 12.1 months among those with a lower proportion (*P* = 0.020, HR [95% CI]: 2.30 [1.12-4.71]) (Figure 4B).

Current treatment regimens for CRC patients mainly involve chemotherapy, either as monotherapy or combined with bevacizumab. Considering the relatively high prevalence of alterations in *KMT2A*-related genes and in the HR pathway, further analyses were performed to examine their association with prognosis in patients undergoing these two treatment options.

**Figure 5:**
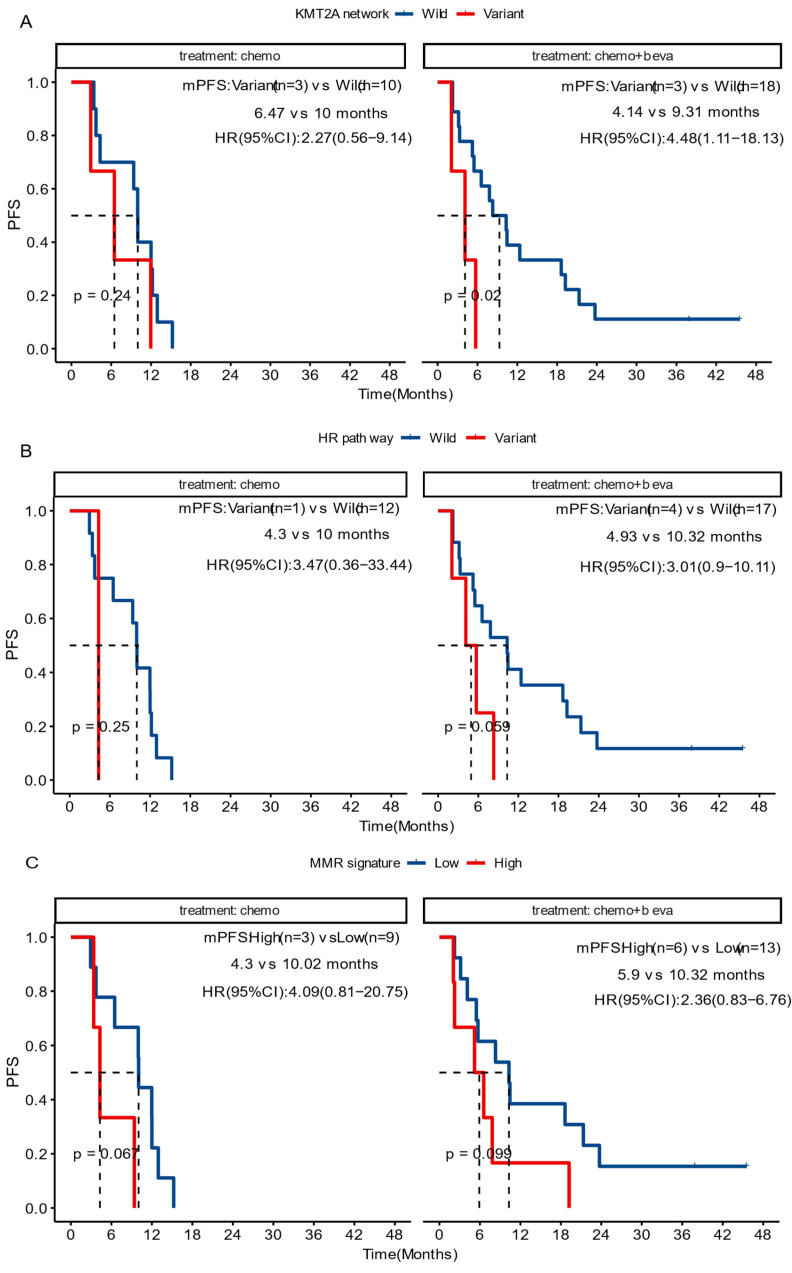
**Prognosis among diverse treatment cohorts.** The impact of variations in the *KMT2A* network (A) and in the HR pathway (B) genes, and the impact of dMMR mutation signature (C), on the progression-free survival of patients receiving chemotherapy (chemo) or chemotherapy plus bevacizumab (chemo+beva).

In the group receiving chemotherapy plus bevacizumab, patients who harbored *KMT2A*-related mutations had a worse prognosis compared to those with the wild-type genes (mPFS 4.1 vs. 9.3 months,* P* = 0.02, HR [95% CI]: 4.48 [1.11-18.13]), and no significant difference was observed in the group receiving chemotherapy alone (mPFS 6.5 vs. 10.0 months, *P* = 0.24, HR [95% CI]: 2.27 [0.56-9.14]) (Figure 5A). Similarly, patients with HR pathway mutations in the combined therapy group had a shorter PFS (mPFS 4.9 vs. 10.9 months, *P* = 0.029, HR [95% CI]: 3.01 [0.9-10.11]), while no significant difference was found in the chemotherapy group (mPFS 4.3 vs. 10.0 months, *P* = 0.25, HR [95% CI]: 3.47[0.36-33.44]) (Figure 5B). We also analyzed the impact of dMMR-related signatures on patient prognosis in these two groups, where we found that patients with a higher proportion of dMMR signatures had a worse prognosis in both groups (Figure 5C). We used Fisher's exact test to investigate the interaction of above-mentioned prognosis-related factors, and found significant co-occurrences between mutations in *KMT2A* and those in genes that belong to its signaling network, as well as between *BRCA2* mutations and the HR pathway mutations (Figure S2). Subsequently we performed a multivariate Cox analysis incorporating relevant clinical and genomic features. Considering the co-occurrences, we selected the *KMT2A* and *BRCA2* mutations, which showed stronger correlation with prognosis in univariate analysis, to represent other mutations in their respective pathways. The multivariate analysis identified three independent prognostic factors associated with PFS: maintenance treatment (HR, 0.22[95% CI, 0.08-0.62]; P =0.004), *KMT2A* mutations (HR, 4.47[95% CI, 1-19.93]; P =0.050), and dMMR-related mutational signatures (HR, 3.57[95% CI, 1.42-8.96]; P =0.007) (Table 2). We further examined the association between molecular mutations such as KMT2A and clinical variables and found no significant association (Table S1).

### External Validation on *KMT2A* Mutation and dMMR Signature

**Figure 6:**
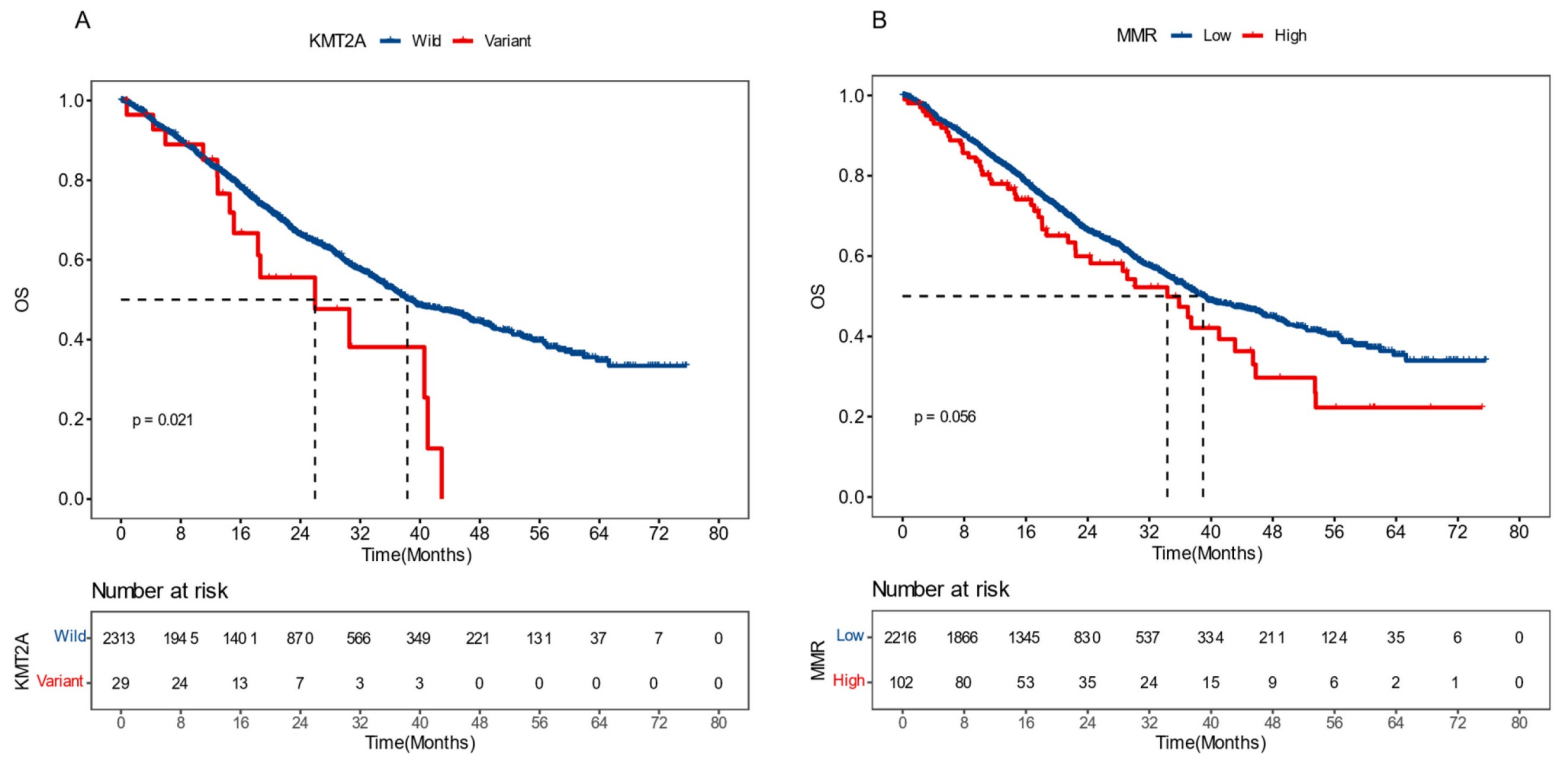
**External validation results of KMT2A gene and dMMR signature.** The influence of *KMT2A* mutation (A) and proportion of dMMR signature (B) on patients' overall survival, derived from the Memorial Sloan Kettering Cancer Center dataset 23.

To validate the prognostic effects of *KMT2A* and dMMR signature in advanced CRC, we gathered data from a 2022 study on pan-cancer metastasis mechanisms by the Memorial Sloan Kettering Cancer Center (MSKCC). The dataset included the mutational and survival information of 2,342 CRC patients who developed distant metastasis and exhibited microsatellite stability (MSS) 23. We demonstrated that, in this dataset, patients with *KMT2A* mutations had significantly shorter overall survival (OS), with a median OS (mOS) of 26.0 months versus 38.3 months for those without (*P* = 0.021, HR [95% CI]: 1.81 [1.08-3.01]) (Figure 6A). Meanwhile, patients with a higher proportion of dMMR mutational signature had marginally worse OS, with a mOS of 34.3 months compared to 39.0 months in patients with a lower proportion (*P* = 0.056, HR [95% CI]: 1.33 [0.99-1.78]) (Figure 6B). These findings further supported the significance of *KMT2A* mutation and dMMR signature in predicting the prognosis of CRC patients with distant metastasis and MSS.

## Discussion

Our study on advanced CRC patients identified several factors potentially predicting a poor prognosis. These include absence of maintenance therapy, genomic variations in *KMT2A* and *BRCA2* and their respective pathways, and enrichment of dMMR-associated mutational signatures. The multivariate Cox analysis revealed that maintenance therapy*, KMT2A* mutations and dMMR mutational signatures remained significantly associated with prognosis, suggesting their potential as independent prognostic indicators. External validation in advanced CRC patients from the MSKCC dataset further supported the negative correlation of *KMT2A* mutations or dMMR signature enrichment with prognosis 23.

The *KMT2* gene family plays a crucial role in epigenetic regulation, with *KMT2A* specifically first found to be involved in the rearrangement of chromosome 11q23 in mixed-lineage leukemia (MLL) 24, 25. Recent genomic sequencing studies have shown that *KMT2* genes are commonly mutated in various human cancers 26. Further studies have revealed that overexpression of KMT2A is associated with an unfavorable prognosis in CRC. KMT2A mediates the interaction of β-catenin with consensus DNA sequences and the subsequent transcription of β-catenin targets, which consequently promotes tumor growth 27. A study conducted by Yang Fang and colleagues revealed an overexpression of KMT2A in CRC tissues compared with paired paracancerous tissues, with the expression level positively correlated with tumor staging. Moreover, *KMT2A*-knockout cells exhibited suppressed cell migration and invasion 28. In contrast, Cun Liao *et a*l. discovered that *KMT2* variation was associated with increased tumor mutational burden (TMB) and MSI, ultimately resulting in improved survival for CRC patients 29. In comparison to prior studies, we scrutinized the KMT2A protein network via the STRING database and demonstrated that mCRC patients with alterations in KMT2A-related genes also exhibited shorter PFS. We further specified that significant PFS difference was only seen in patients receiving chemotherapy plus bevacizumab, but not in those receiving chemotherapy alone. These findings emphasize the need for a comprehensive understanding of the KMT2A network and its role in tumor progression and drug response, especially to bevacizumab, a frequently used therapeutic agent in CRC. Further studies remain to explore the potential of targeting KMT2A and its interacting partners as a novel therapeutic strategy for CRC.

Extensive research has been conducted on the prevalence and impact of *BRCA* mutations in cancers. Initial studies have shown that individuals with *BRCA1* or *BRCA2* mutations were 84% more susceptible to breast cancer and 40% more susceptible to ovarian cancer throughout their lifetime 30-32. *BRCA1*/*BRCA2* mutations have also been linked to increased risks of prostate, pancreatic, and gastric cancers 33, 34. A meta-analysis has suggested that individuals with *BRCA1* mutations have a higher risk of developing CRC, but the risk is not higher for those with *BRCA2* mutations; However, the findings of this study are subject to debate 35. Studies have also indicated that CRC patients with *BRCA* mutations are more likely to benefit from chemotherapy. However, this conclusion was primarily based on case reports and limited retrospective case-control analyses that mainly concerned chemotherapy regimens involving oxaliplatin 36, 37. In our study, we revealed that mCRC patients with *BRCA2* mutations had a worse prognosis than those without. Treatment outcomes were also suboptimal for patients harboring HR pathway mutations, especially in the cohort receiving chemotherapy plus bevacizumab. HR has been identified as a crucial DNA repair mechanism in mammalian cells, and the indispensable role of the tumor suppressors BRCA1 and BRCA2 in HR has been well established 38, 39. HR deficiency is now considered targetable in cancer treatment as it renders tumor cells more susceptible to certain DNA-damaging agents 40, 41.

The development of CRC is influenced not only by single mutations but also by the combined effect of multiple factors, which yield various genomic patterns termed mutational signatures 42. An algorithm has been developed to quantify the differential contributions of these mutation signatures to genomic alteration, by assigning them each an activity level, or “exposure” 43. This provides a more precise understanding of the tumor's genomic feature and the processes underlying its development. Various types of DNA repair mechanisms are activated in response to either endogenous or exogenous DNA damage 44. The MMR mechanism, in particular, is responsible for correcting base pair mismatches that occur during DNA replication 45 and serves as a physiological barrier against genomic instability and the accumulation of somatic mutations 46. However, genetic and epigenetic alterations can disrupt these DNA repair pathways, which can be exploited by tumor cells. Consequently, mutations accumulate and their pattern can be recognized by mutational signature analysis 47. For example, MSI is almost always a direct result of MMR malfunction 48. The close association between MMR pathway deficiency and both sporadic and hereditary CRC cases, including Lynch syndrome, is well-established 45, 49, 50. In this study, we found that mutational signatures of the studied mCRC population were primarily determined by age, dMMR, and APOBEC. Additionally, we established a threshold for the proportion of dMMR-related signatures and discovered that patients with a higher proportion tended to have a poorer prognosis. This finding has also been validated by external data. Previous studies have indicated that MSI is considered a favorable prognostic factor and a strong negative predictive factor for 5-fluorouracil therapy in stage II colon cancer 6. However, in cases of metastasis, the impact of MSI on survival is still controversial, with conflicting results reported in studies within this area.

In summary, our investigation has underscored the importance of *KMT2A* mutations and dMMR mutational signatures as independent prognostic markers in mCRC. However, we acknowledge the limitations inherent in our study, particularly regarding the size of our cohort. The possibility of data overinterpretation cannot be overlooked, and we emphasize the necessity of further validation within larger and independent patient cohorts to solidify these preliminary insights.

## Supplementary Material

Supplementary figures and tables.

## Figures and Tables

**Table 1:**
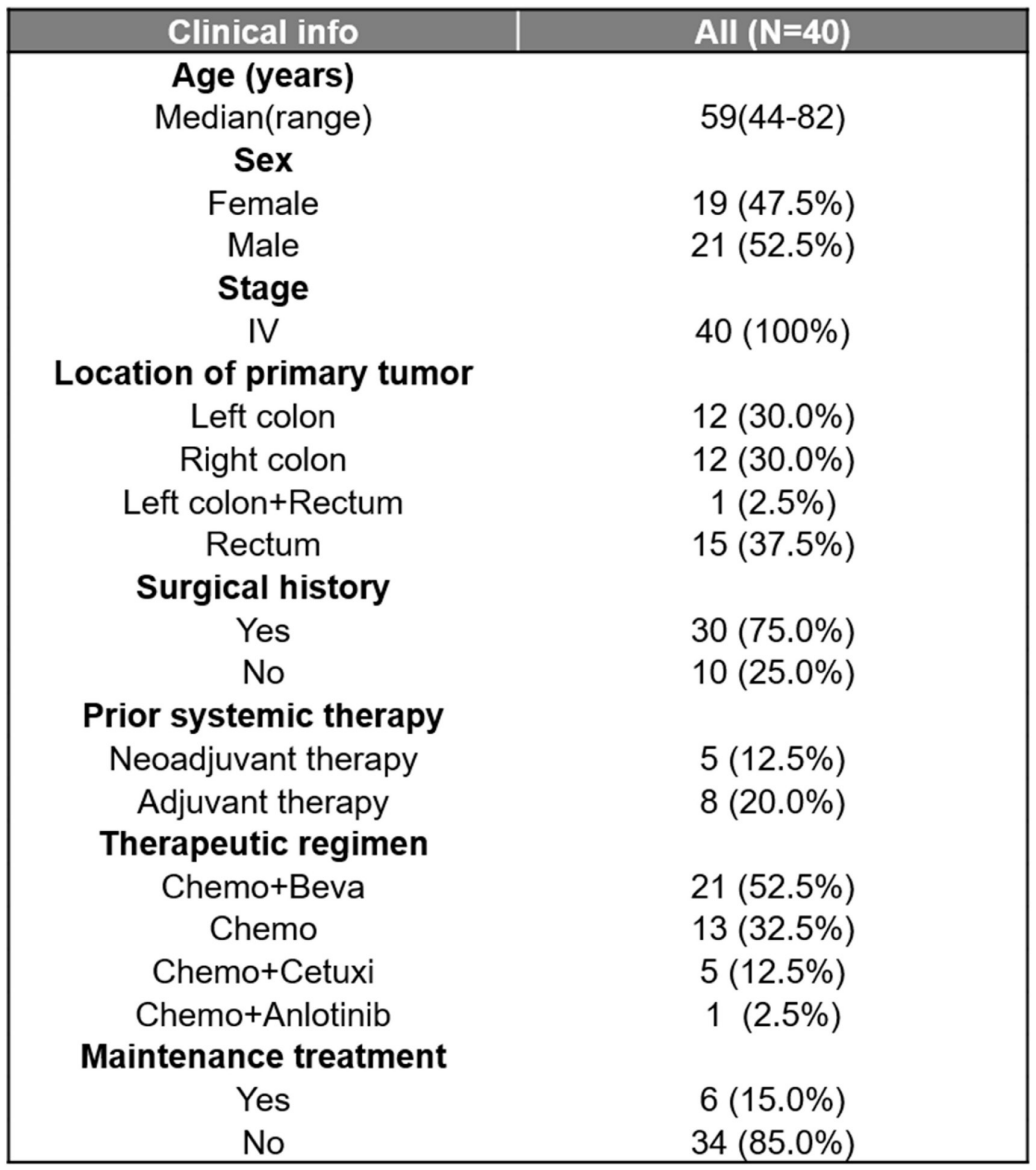
Clinical characteristics of the enrolled CRC patients who had baseline tissue samples (N=40).

Beva, bevacizumab; Cetuxi, cetuximab; Chemo, chemotherapy.

**Table 2:**
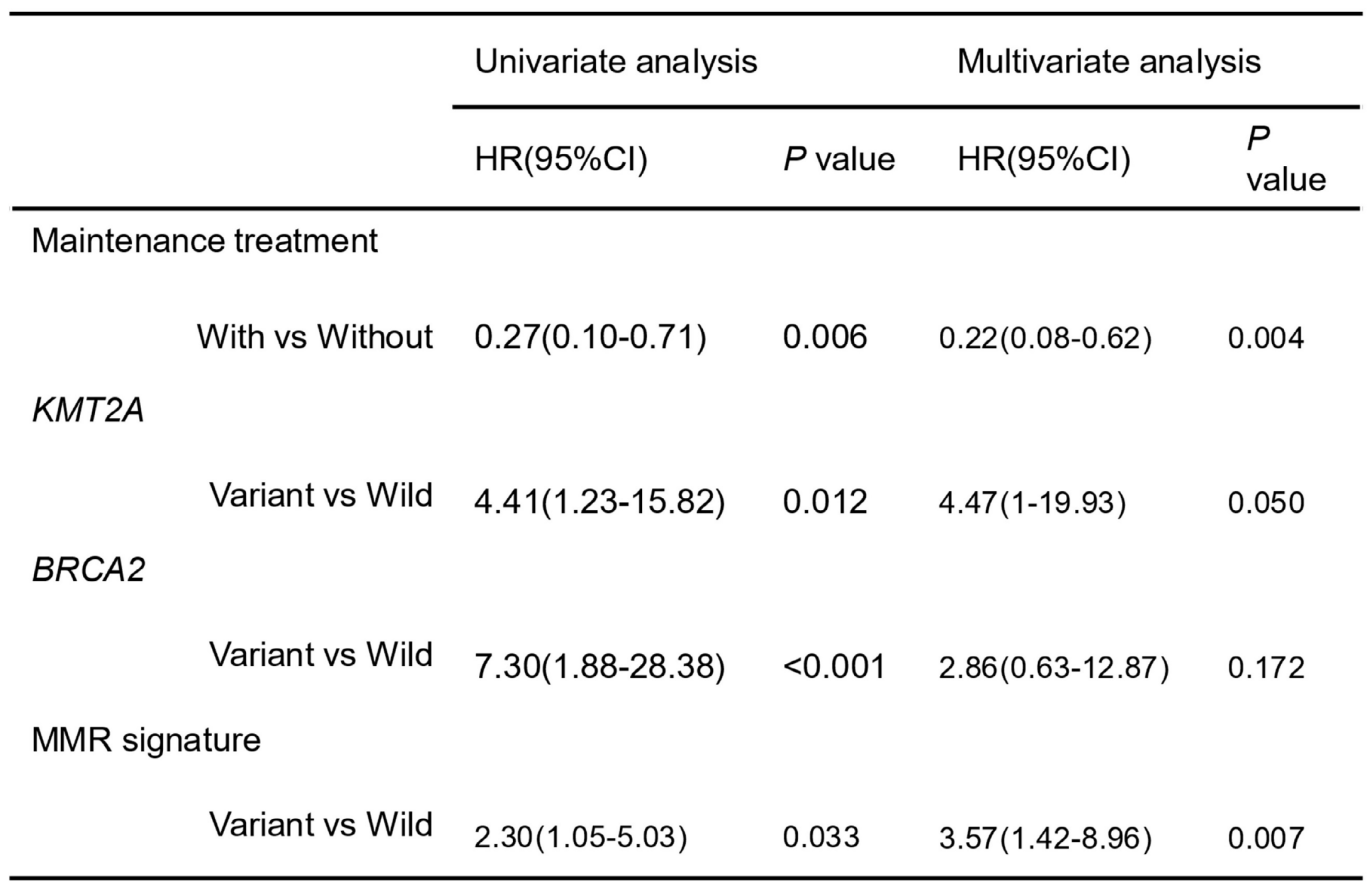
Cox multivariate analysis of clinical and genomic features affecting patients' progression-free survival.

CI, confidence interval; HR, hazard ratio.
