# Supplementary material for: KMT2A Mutations and High Prevalence of dMMR-associated Mutational Signatures as Prognostic Indicators in Metastatic Colorectal Cancer
Source: J Cancer. 2024 Apr 8;15(10):3140–50. doi: 10.7150/jca.94410 (PMC11064249; doi:10.7150/jca.94410)

Supplementary materials.

Figure S1 Patient enrollment flowchart

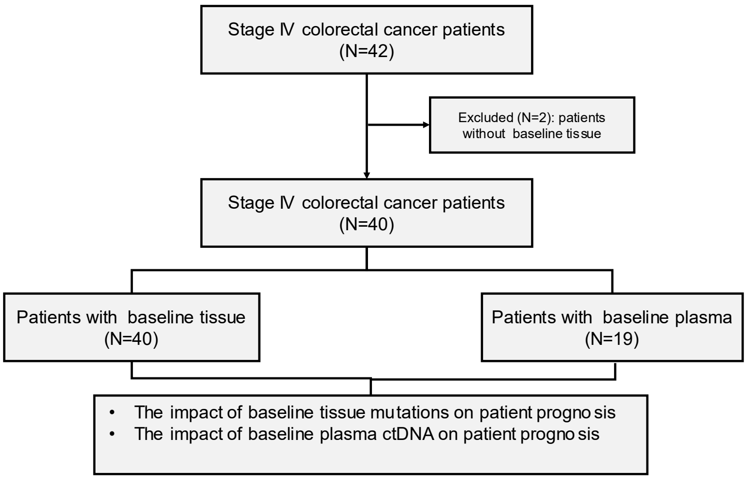

Table S1

KMT2A	KMT2A Wild (N=37)	KMT2A Variant (N=3)	*P* value
**Age (years)**			>0.999
≤59	21 (56.8%)	2 (66.7%)	
>59	16 (43.2%)	1 (33.3%)	
**Sex**			0.233
Female	19 (51.4%)	0 (0%)	
Male	18 (48.6%)	3 (100%)	
**Location of primary tumor**			>0.999
Left colon	11 (29.7%)	1 (33.3%)	
Left colon+Rectum	1 (2.7%)	0 (0%)	
Rectum	14 (37.8%)	1 (33.3%)	
Right colon	11 (29.7%)	1 (33.3%)	
**Surgical history**			>0.999
Yes	28 (75.7%)	2 (66.7%)	
No	9 (24.3%)	1 (33.3%)	
**Prior systemic therapy**			
**Neoadjuvant therapy**			>0.999
Yes	5 (13.5%)	0 (0%)	
No	32 (86.5%)	3 (100%)	
**Adjuvant therapy**			0.096
Yes	6 (16.2%)	2 (66.7%)	
No	31 (83.8%)	1 (33.3%)	
**Maintenance treatment**			>0.999
Yes	6 (16.2%)	0 (0%)	
No	31 (83.8%)	3 (100%)	
**Therapeutic regimen**			0.724
Chemo	11 (29.7%)	2 (66.7%)	
Chemo+Anlotinib	1 (2.7%)	0 (0%)	
Chemo+Beva	20 (54.1%)	1 (33.3%)	
Chemo+Cetuxi	5 (13.5%)	0 (0%)	

KMT2A network	KMT2A network Wild (N=33)	KMT2A network Variant (N=7)	*P* value
**Age (years)**			>0.999
≤59	19 (57.6%)	4 (57.1%)	
>59	14 (42.4%)	3 (42.9%)	
**Sex**			
Female	15 (45.5%)	4 (57.1%)	
Male	18 (54.5%)	3 (42.9%)	
**Location of primary tumor**			0.894
Left colon	10 (30.3%)	2 (28.6%)	
Left colon+Rectum	1 (3.0%)	0 (0%)	
Rectum	13 (39.4%)	2 (28.6%)	
Right colon	9 (27.3%)	3 (42.9%)	
**Surgical history**			>0.999
Yes	25 (75.8%)	5 (71.4%)	
No	8 (24.2%)	2 (28.6%)	
**Prior systemic therapy**			
**Neoadjuvant therapy**			>0.999
Yes	4 (12.1%)	1 (14.3%)	
No	29 (87.9%)	6 (85.7%)	
**Adjuvant therapy**			0.611
Yes	6 (18.2%)	2 (28.6%)	
No	27 (81.8%)	5 (71.4%)	
**Maintenance treatment**			0.567
Yes	6 (18.2%)	0 (0%)	
No	27 (81.8%)	7 (100%)	
**Therapeutic regimen**			0.875
Chemo	10 (30.3%)	3 (42.9%)	
Chemo+Anlotinib	1 (3.0%)	0 (0%)	
Chemo+Beva	18 (54.5%)	3 (42.9%)	
Chemo+Cetuxi	4 (12.1%)	1 (14.3%)	

MMR signature	MMR signature ratio Low (N=28)	MMR signature ratio High (N=9)	*P* value
**Age (years)**			0.438
≤59	18 (64.3%)	4 (44.4%)	
>59	10 (35.7%)	5 (55.6%)	
**Sex**			0.703
Female	12 (42.9%)	5 (55.6%)	
Male	16 (57.1%)	4 (44.4%)	
**Location of primary tumor**			0.919
Left colon	8 (28.6%)	3 (33.3%)	
Left colon+Rectum	1 (3.6%)	0 (0%)	
Rectum	12 (42.9%)	3 (33.3%)	
Right colon	7 (25.0%)	3 (33.3%)	
**Surgical history**			>0.999
Yes	21 (75.0%)	7 (77.8%)	
No	7 (25.0%)	2 (22.2%)	
**Prior systemic therapy**			
**Neoadjuvant therapy**			0.307
Yes	5 (17.9%)	0 (0%)	
No	23 (82.1%)	9 (100%)	
**Adjuvant therapy**			0.656
Yes	6 (21.4%)	1 (11.1%)	
No	22 (78.6%)	8 (88.9%)	
**Maintenance treatment**			>0.999
Yes	5 (17.9%)	1 (11.1%)	
No	23 (82.1%)	8 (88.9%)	
**Therapeutic regimen**			0.618
Chemo	9 (32.1%)	3 (33.3%)	
Chemo+Anlotinib	1 (3.6%)	0 (0%)	
Chemo+Beva	13 (46.4%)	6 (66.7%)	
Chemo+Cetuxi	5 (17.9%)	0 (0%)	

FigureS2 Correlation analysis of clinical and genetic features related to prognosis

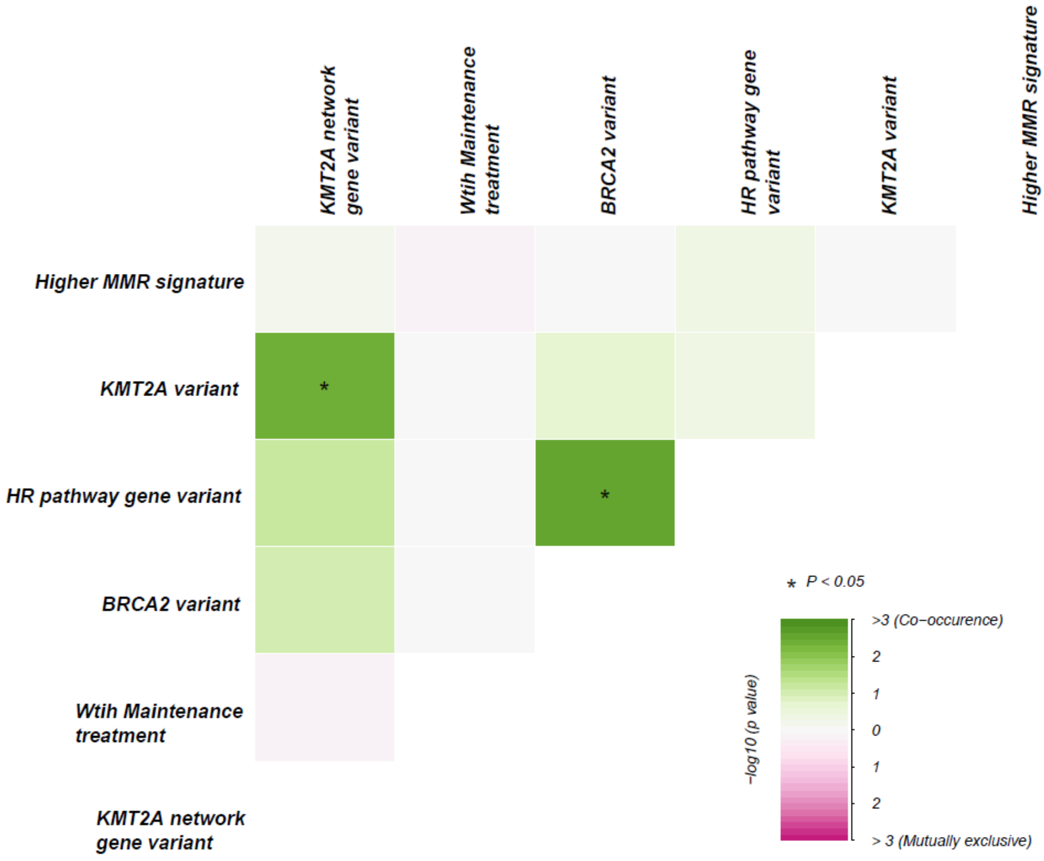

Supplement: Supplementary file 1 — Supplementary figures and tables. [file jcav15p3140s1.pdf]
